# Vacuum Spin LED: First Step towards Vacuum Semiconductor Spintronics

**DOI:** 10.3390/nano13030422

**Published:** 2023-01-19

**Authors:** Oleg E. Tereshchenko, Vladimir A. Golyashov, Vadim S. Rusetsky, Danil A. Kustov, Andrey V. Mironov, Alexander Yu. Demin

**Affiliations:** 1 Rzhanov Institute of Semiconductor Physics, Siberian Branch, Russian Academy of Sciences, Novosibirsk 630090, Russia; 2 Synchrotron Radiation Facility SKIF, Boreskov Institute of Catalysis, Siberian Branch, Russian Academy of Sciences, Kol’tsovo 630559, Russia; 3 CJSC “Ekran FEP”, Novosibirsk 630060, Russia

**Keywords:** electrons, spin, spintronics, quantum wells, spin LED, optical orientation, negative electron affinity

## Abstract

Improving the efficiency of spin generation, injection, and detection remains a key challenge for semiconductor spintronics. Electrical injection and optical orientation are two methods of creating spin polarization in semiconductors, which traditionally require specially tailored p-n junctions, tunnel or Schottky barriers. Alternatively, we introduce here a novel concept for spin-polarized electron emission/injection combining the optocoupler principle based on vacuum spin-polarized light-emitting diode (spin VLED) making it possible to measure the free electron beam polarization injected into the III-V heterostructure with quantum wells (QWs) based on the detection of polarized cathodoluminescence (CL). To study the spin-dependent emission/injection, we developed spin VLEDs, which consist of a compact proximity-focused vacuum tube with a spin-polarized electron source (p-GaAs(Cs,O) or Na_2_KSb) and the spin detector (III-V heterostructure), both activated to a negative electron affinity (NEA) state. The coupling between the photon helicity and the spin angular momentum of the electrons in the photoemission and injection/detection processes is realized without using either magnetic material or a magnetic field. Spin-current detection efficiency in spin VLED is found to be 27% at room temperature. The created vacuum spin LED paves the way for optical generation and spin manipulation in the developing vacuum semiconductor spintronics.

## 1. Introduction

A promising trend in the development of modern electronics is so-called “vacuum microelectronics” [1,2], now more commonly referred to as “vacuum nanoelectronics” [3]. This term is used to describe devices or components possessing micro- and nanometer-scale geometric dimensions whose principle of operation is based on the phenomenon of field electron emission [3]. Over the past few decades, vacuum semiconductor nanoelectronics have appeared to be a promising area for the development of high-speed and radiation-resistant integrated circuits [4]. Based on the idea of the general development of semiconductor technology, it can be assumed that future developments of vacuum nanoelectronics will include “*vacuum spintronics*” or “*vacuum semiconductor spintronics*”.

**Figure 1:**
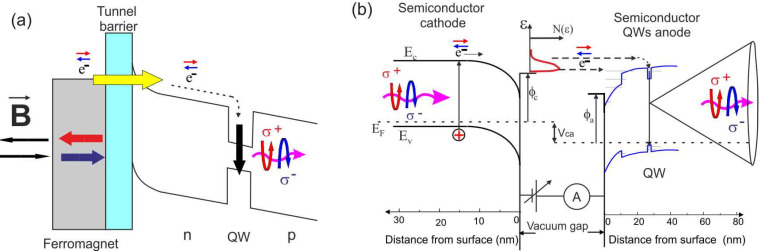
Transformation of monolithic spin LED (**a**) into two electrodes (anode and cathode) with the NEA states separated by the vacuum gap (**b**). Spin-polarized electrons emitted from the cathode and injected to the anode through the vacuum gap. In the case of a monolithic spin LED, an FM contact (or/and a magnetic semiconductor) and an external magnetic field are required to switch between polarizations, whereas a vacuum spin LED uses only the optical pumping effect to create, inject, and detect the spin polarization.

One of the most important requirements for spintronic devices is the efficient injection of spin-polarized carriers. Solid-state monolithic spin LED structures open the way to studying spin injection [5]. Spin-polarized electrons (holes) can be injected into a semiconductor by flowing electrical current from a ferromagnetic (FM) material (Figure 1a). In spin LED, spin-polarized carriers injected from the FM metals radiatively recombine in semiconductor (SM) emitting circularly polarized (CP) light. The injection efficiency depends on the spin polarization of the FM, the spin scattering at the FM/NM interface and requires a high resistance interface contact. There are two ways to satisfy this limitation: implementing a metal–oxide tunnel barrier or a Schottky contact. Successful results were obtained for both approaches using GaAs-based spin LEDs [6,7]. It was found that tunneling or Schottky contacts between a metallic FM and a SC can overcome the conductance mismatch obstacle and show carrier polarizations up to 30% [6,8]. Recently, it has also been found that spin signal is also determined by a dynamic factor arising from the competition between tunneling into the FM and recombination with the holes [9]. With coherent tunneling using the MgO barrier, a further increase in efficiency to 100% was predicted, albeit only theoretically [10,11].

One of the solutions is to use a source and an injector (detector) of spin-polarized electrons mechanically separated by a vacuum gap. Since interfaces have been shown to be crucial in spin-dependent tunnel experiments [12], a quantitative comparison of injection across the vacuum gap should provide fundamental and practical insight into the spin injection and spin relaxation processes. Vacuum is inherently superior to solid state as a medium for carrier transport since it allows ballistic transport, while carriers suffer from phonons, and defects scattering in semiconductors and the conduction mismatch obstacle is naturally overcome as well.

Transformation of a monolithic spin LED based on FM Schottky contact and p-n junction into two independent electrodes (cathode and anode) separated by the vacuum gap is shown in Figure 1. To transfer spin-polarized electrons from a cathode to an anode, the work function of both electrodes should be reduced. For semiconductors, reducing the work function has traditionally been achieved by coating surfaces with cesium and oxygen (Cs, O). [13,14] or (Cs, Sb) [15]. The p-type cathode with (Cs, O) layer produces the so-called effective negative electron affinity state (NEA) (vacuum level is below the conduction band maximum in the bulk) (Figure 1b), which makes it possible to reach a quantum efficiency greater than 50% for photocathodes such as GaN, GaAs and GaAsP [16,17]. Moreover, based on both the phenomenon of optical spins orientation in semiconductors (generation of spin-oriented electrons with circularly polarized light absorption) and the discovery of NEA, the GaAs as a spin-polarized source was realized [18,19].

The process of injection into a semiconductor structure is inverse to the emission of electrons from the semiconductor because of the time-reversed nature of the Maxwell and Schrödinger equations. The spin-polarized electrons injected into the III-V heterostructure recombine with holes and produce polarized cathodoluminescence emission. Thus, spin-polarized electrons are emitted from the cathode to the anode through the vacuum gap. An important consequence of the separation of the emitter and the detector by a vacuum gap is the independence of the choice of electrode material. As mentioned above, the vacuum provides ballistic transport without carrier scattering, but what do we know about spin scattering during emission? For III-V photocathodes, it has been convincingly demonstrated that spin polarization of the emitted electrons can reach a value tending to be very close to the theoretical limit of 49% for GaAs [20,21] and above 90% for the strained III-V superlattice [22]. High spin polarization of electrons photoemitted from III-V photocathodes confirms the neglect of spin scattering at the well-prepared cathode/vacuum interface. It can be assumed that when injecting electrons into a semiconductor from a vacuum, spin scattering at a well-prepared vacuum/anode interface can also be neglected [23]. This provides a unique opportunity to study the mechanisms of spin relaxation depending on the energy of the injected electrons into the target.

We fabricated a combined vacuum photodiode and spin LED detector designed to provide efficient vacuum barrier injection of spin-polarized electrons from GaAs or Na_2_KSb photocathode sources into an Al_x_Ga_1−x_As and Al_x_Ga_1−x_As/GaAs quantum well (QW) LED structure. Spin polarization of free electrons injected into SC is detected optically through circularly polarized CL from the SC. Such structures can be referred to as spin-polarized vacuum light-emitting diodes (spin VLEDs), which operate without the use of magnetic material or a magnetic field; generation and detection of spin-polarized electrons are based only on the optical spin pumping effect, which ensures the generation of spin-polarized carriers without the need for charge currents. A spin-polarized electron transmitted through the vacuum was detected as CP cathodoluminescence (CL) signals, which are in the range of 2–10% at room temperature (RT) for different structures. This value corresponds to the 20–50% of minority electron spin polarization in the conduction band of the photocathode. The injection of polarized low-energy electrons into an SC target was investigated by varying the kinetic energy in the range of 0.5–5.0 eV and the temperature in the range of 10–300 K. This made it possible to propose a new type of semiconductor spin detector for free electrons with spatial resolution [24]. The injection method of polarization detection based on electron–photon conversion is an alternative to spin detection based on spin orbit or exchange interactions.

## 2. Materials and Methods

**Figure 2:**
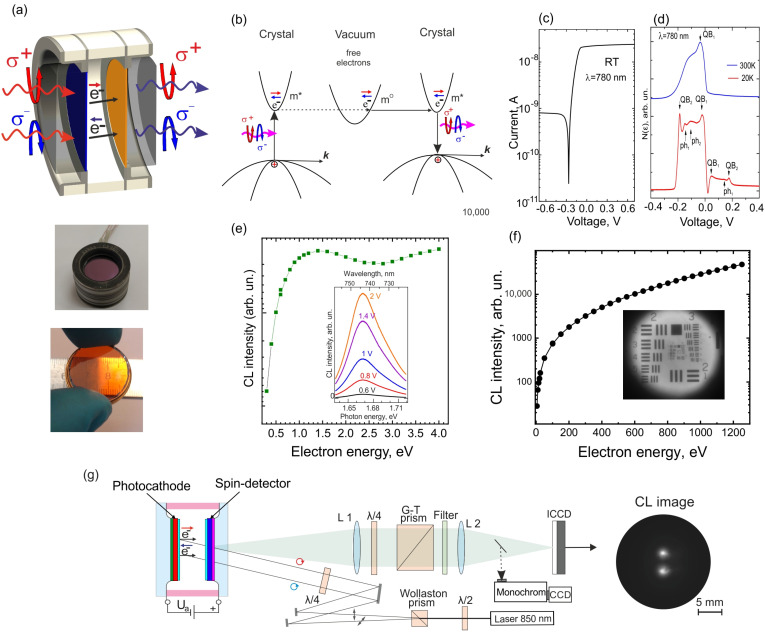
(**a**) Scheme in cross-section of compact vacuum photodiode for studying the emission and injection physics of spin-polarized electrons. Photographs below show the assembled photodiode from the photocathode side and the anode QW structure. The electrons photoemitted from the GaAs (Na_2_KSb) source are injected into the SC heterostructure with QW. (**b**) Schematic band structure of GaAs (Na_2_KSb) cathode and semiconductor anode near the Γ-point illustrating the photoemission process in vacuum and injection in semiconductor structure. m^0^- is the rest mass of an electron and m*- is the effective mass. (**c**) I-V curve illustrating the photodiode characteristic under the illumination with the 780 nm wavelength. (**d**) Normalized energy distribution curves (EDC) at 300 and 20 K. (**e**) Dependence of CL intensity (logarithmic scale) on the electron energy injected into the Al_x_Ga_1−x_As/GaAs/Al_x_Ga_1−x_As heterostructure. The insert shows CL spectra measured at various accelerating biases. (**f**) Similar to (**e**), but measured at higher electron energy. Insert shows the CL image of the test chart detected at 1 keV electron energy by a standard CCD camera. (**g**) Schematic drawing of optical setup for spatial CL polarization detection. Electrons emitted by ℏν-photon excitation from the photocathode (GaAs or Na_2_KSb) source are injected into the spin-detector (QWs or Al_0.11_Ga_0.89_As) target.

The spin-dependent injection and photoemission properties of two semiconductor electrodes with effective NEA were studied in a parallel-plate capacitor-like vacuum photodiode. Figure 2 shows a schematic presentation of the compact vacuum photodiode with photographs of the device from the cathode and the anode (target) sides (Figure 2a) and schematic drawing of optical setup for spatial CL polarization detection (Figure 2g). A more detailed experimental scheme and description of the operation principle for the investigation of spin-dependent injection can be found in Ref. [15]. Figure 2b shows the band structures of two semiconductor electrodes with NEA separated by a vacuum gap and illustrating the photoemission process in vacuum and subsequent injection of spin-polarized electrons in SC structure followed by recombination. Two types of photocathode were used as a source of spin-polarized electrons. The first consisted of a GaAs active layer with a thickness of 2.5 μm and an Al_0.6_Ga_0.4_As layer with a SiO coating bonded to the glass input window (Figure 2a).

The second one consists of an active polycrystalline Na_2_KSb layer grown by vapor phase deposition on the glass of the input window and activated to NEA by Cs_3_Sb layer [15]. The two kinds of SC heterostructures were used as an anode (spin detector). The first, GaAs/AlGaAs QWs structure consists of an upper 10 nm GaAs layer, Al_x_Ga_1−x_As layer with the thickness of 15 nm (5 nm, x = 0.3; 5 nm, x = 0.6; and 5 nm, x = 0.3), three GaAs QWs with a width of 7 ML (2.0 nm) separated by 20 nm Al_0.3_Ga_0.7_As barriers, and the last 100 nm Al_0.6_Ga_0.4_As layer bonded to the output window through the SiO antireflection coating. Both anode and cathode were doped by an acceptor up to concentrations of 3·10^17^ and 6·10^18^ cm^−3^, respectively. The second detector was the 200 nm Al_0.11_Ga_0.89_As layer.

To prepare the NEA state on both the cathode and the anode (QWs), first, the active-layer surface-cleaning procedure was performed in order to remove the native oxides and passivate a surface by elemental arsenic [25]. Then, atomically cleaned Ga-stabilized surfaces [26] were prepared by vacuum annealing and activated to the NEA states by coadsorption of cesium and oxygen [13] in the activation chamber. The cleaned Al_0.11_Ga_0.89_As anode was also activated to the NEA state by coadsorption of cesium and antimony. The photocathode and anode were mounted plane-parallel on the opposite sides of a cylindrical alumina ceramics body.

The main feature of the created vacuum photodiodes is that both electrodes are semiconductor heterostructures with close work functions and the NEA state, as shown in Figure 1b. As a result, a typical diode-type photocurrent voltage characteristic (I-V curve) is shown Figure 2c. One can see that, at V = 0, there is the photocurrent in the vacuum diode, and the open circuit voltage is about 0.3 V. This led us to the idea that a photodiode of this type could be used as a solar energy converter based on a photoemission solar cell [27].

Due to the axial symmetry of the photodiode, illumination can be carried out from both sides, which allows both the quantum yield (QY) spectra and the photoelectron energy distribution (EDC) to be measured along the longitudinal component N(ε), using the photodiode as a retarding field electron spectrometer. EDC provides important information about the electron energy loss mechanisms during the emission. The EDCs of the emitted electrons N(ε) in the transmission mode measured 300 K, while 20 K are shown in Figure 2d. It can be seen that both electrodes contribute to the EDC. The existence of the fine structure (QB1, QB2) in the EDC is associated with the electron–phonon coupling in 2D quantized states in the band-bending region [27].

For electrons injected with energies of about 0.5 eV, it becomes possible to penetrate the semiconductor anode and recombine with holes, thus producing cathodoluminescent light from the primary electrons. The CL spectra detected with different accelerating voltages between the cathode and anode are shown in the inset to Figure 2e. The intensity increases rapidly by several orders of magnitude in the range from 0.5 to 1 eV, which is caused by the injection of electrons into the semiconductor bulk and their subsequent radiative recombination. The CL signal continues to increase strongly when electron energy is increased to 1 keV due to the generation of a cascade of secondary electrons, as shown in Figure 2f [28].

## 3. Results

**Figure 3:**
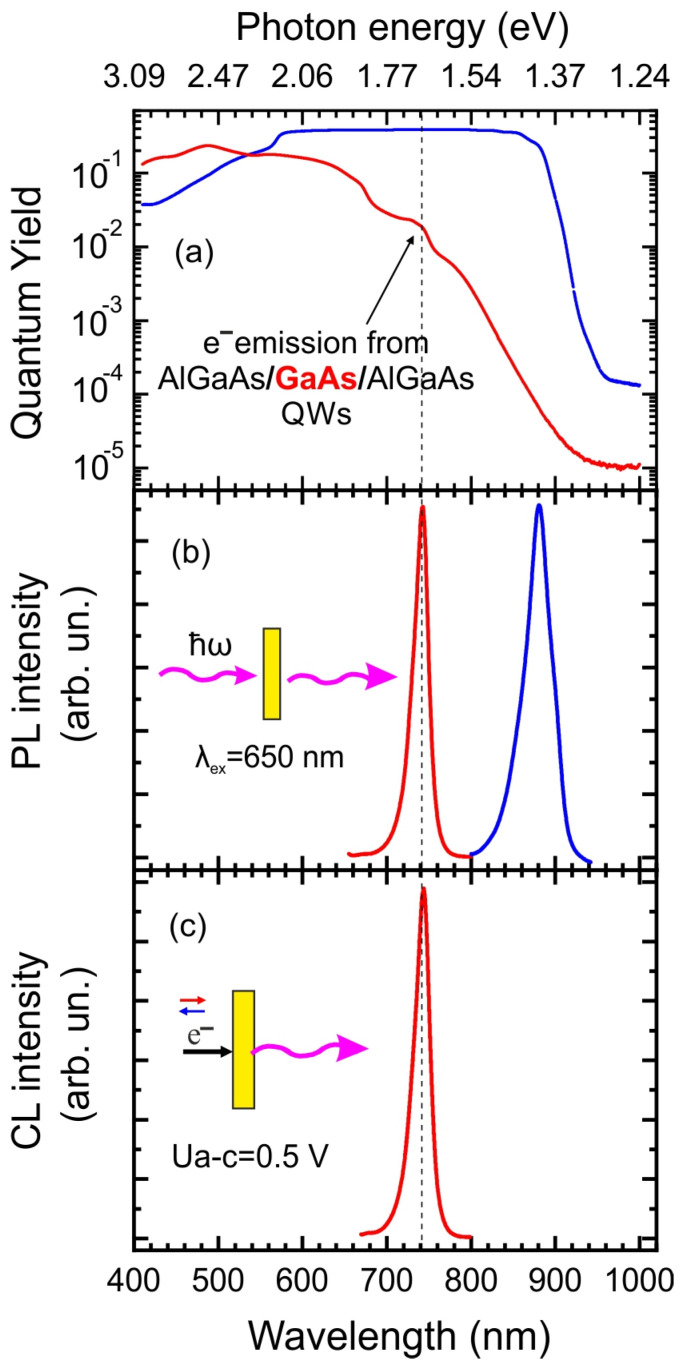
(**a**) Quantum yield (QY) spectra of GaAs photocathode (blue) and Al_x_Ga_1−x_As/GaAs QWs target (red). The QY spectra were measured by applying of 1 V to the emission electrode. The arrow denotes the feature corresponding to the electron emission from quantum-dimensional (QD) levels. (**b**) Photoluminescence spectra from GaAs photocathode (blue) and Al_x_Ga_1−x_As/GaAs QWs target (red). (**c**) Cathodoluminescence spectrum of an Al_x_Ga_1−x_As/GaAs QWs target excited by injection of electrons photoemitted from the photocathode at an accelerating voltage of 0.5 V. The peak positions in the photoemission spectra (**a**), PL (**b**) and CL (**c**) have the same energy and comply with the calculated QD levels in QWs.

In the first stage, we studied the photoemission and injection properties of our photodiodes. The QY spectra for a standard GaAs photocathode and the GaAs/Al_0.3_Ga_0.7_As heterostructure with QWs are shown in Figure 3a. Since both electrodes are bonded to the glass, this allows measuring the QY for the cathode and anode. Similar to the GaAs cathode with NEA, the heterostructure with QWs activated to NEA also shows the properties of a photoemitter. One can see that the QY spectrum of QWs heterostructure shows a specific feature at 742 nm (1.67 eV) in contrast to the spectrum of a typical GaAs photocathode (Figure 3a). The energy position of this peak in the photoemission spectrum, according to calculations, corresponds to the emission from the quantum-dimensional (QD) level in QWs and correlates well with the peak positions in the photoluminescence (PL) and CL spectra shown in Figure 3b and Figure 3c, respectively.

**Figure 4:**
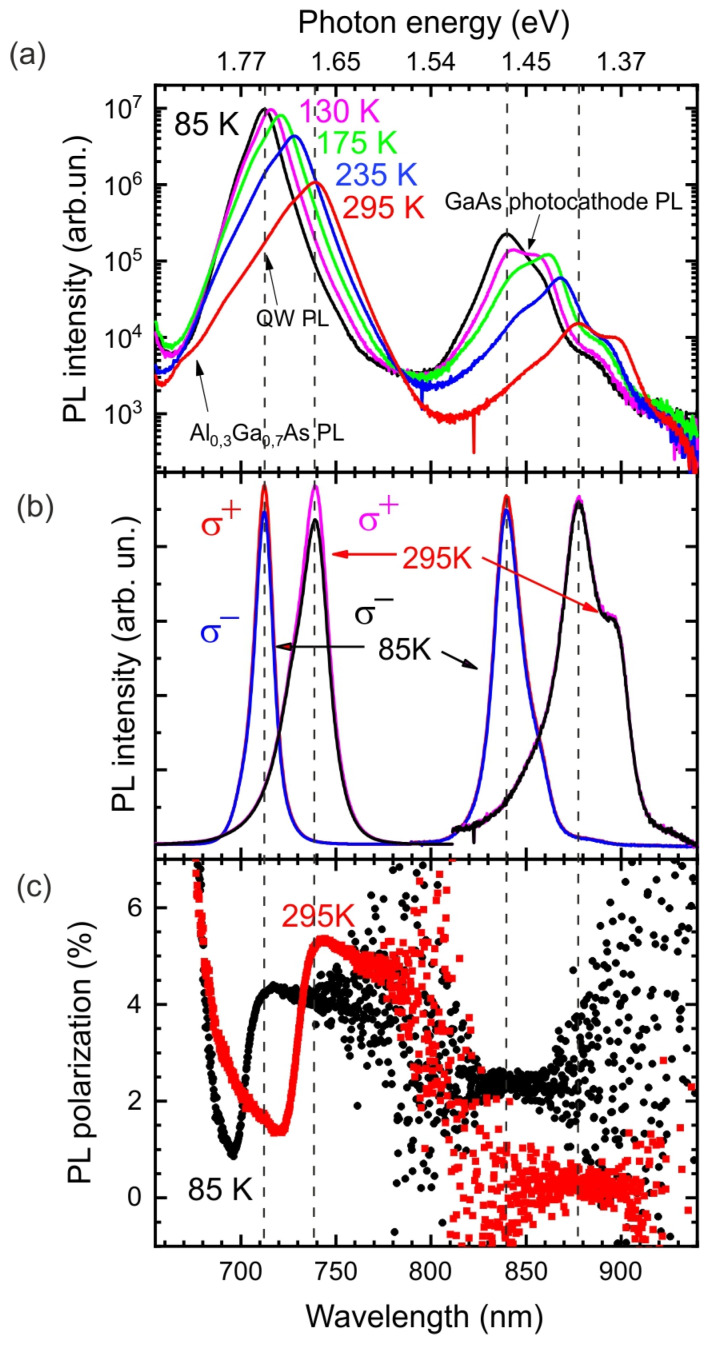
(**a**) Photoluminescence spectra of the cathode and anode recorded at various temperatures. (**b**) Circularly polarized (CP) components (σ+, σ−) of the PL spectra of GaAs/Al_x_Ga_1−x_As QWs and GaAs photocathode measured at 85 and 300 K. (**c**) PL polarization of GaAs/Al_x_Ga_1−x_As QWs and GaAs photocathode. PL of both electrodes was excited with circular polarized emission from a laser diode (650 nm (1.91 eV)).

In the second stage, the polarization properties of the detector and cathode were tested by photoluminescence using the optical pumping effect. In order to gain insight into recombination and spin relaxation processes, the circularly polarized (CP) photoluminescence spectra (*σ*+, *σ*−) of GaAs/Al_x_Ga_1−x_As anode QWs and GaAs photocathode were detected in an optical orientation mode. The PL spectra of the cathode and anode recorded at various temperatures are shown in Figure 4a. One can see that decreasing the temperature to 85 K increases the photoluminescence intensity by an order of magnitude, which is associated with a decrease in the rate of emission-free recombination.

The polarized PL spectra and the degree of PL polarization (P_PL_) at 85 and 295 K are shown in Figure 4b and Figure 4c, respectively. It is well established that P_PL_ is determined by the *τ_r_/τ_s_* ratio temperature dependence, where *τ_r_* and *τ_s_* are the lifetime and spin relaxation time of the electron in the QW. For the GaAs/Al_x_Ga_1−x_As QWs with a thickness less than 10 nm τ_s_ is independent of temperature [29]. Therefore, a decrease in the degree of PL polarization with temperature decrease is caused by an increase in the lifetime *τ_r_*.

**Figure 5:**
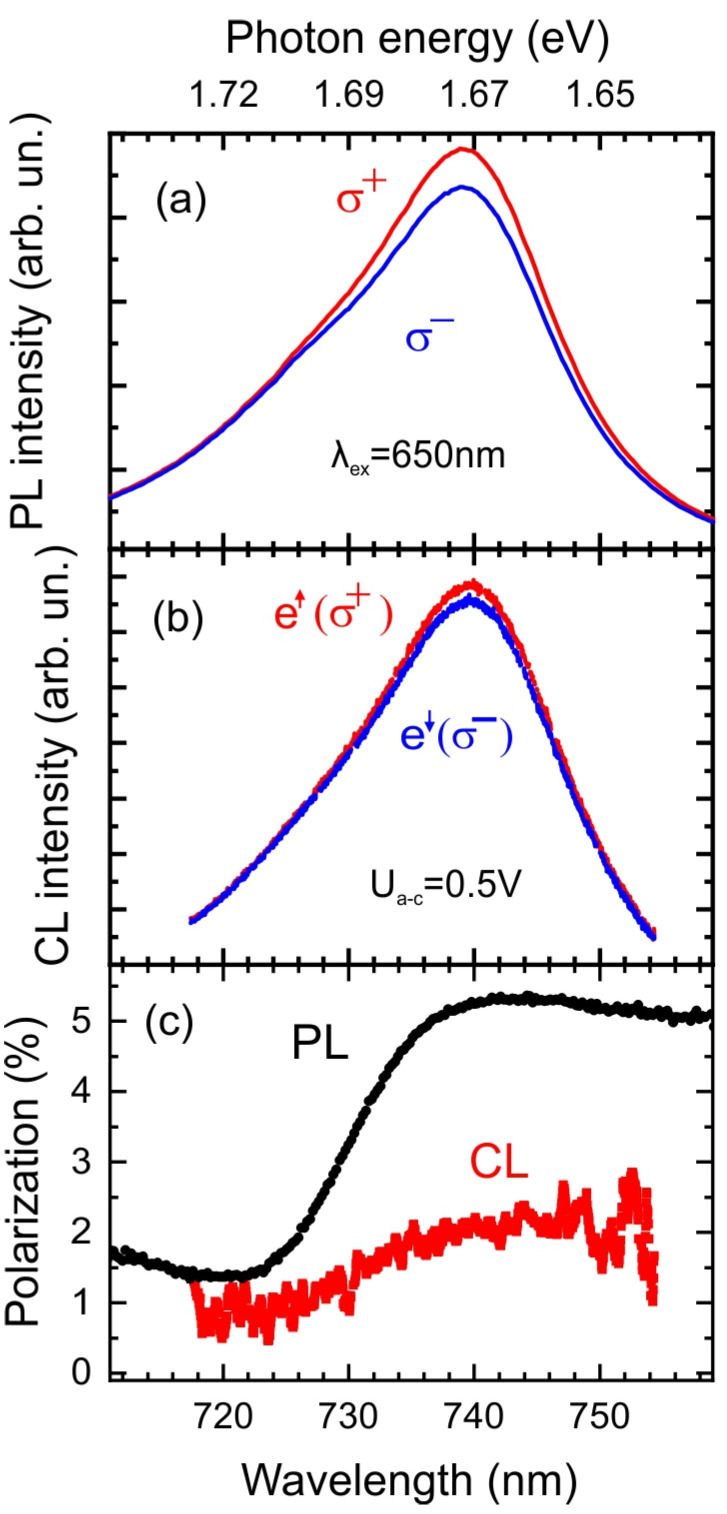
(**a**) PL circularly polarized components (σ+, σ−) spectrum of GaAs/Al_x_Ga_1−x_As QWs excited by the CP emission of laser diode (650 nm). (**b**) CP components (σ+, σ−) of the CL spectra from injected spin-polarized electrons at the accelerating voltage of 0.5 V at 300 K. (**c**) The CP degree of the PL and CL emission at room temperature.

Circularly polarized components (σ+, σ−) of the PL and CL spectra of GaAs/Al_x_Ga_1−x_As anode QWs were detected in the mode of optical orientation and shown in Figure 5a and Figure 5b, respectively. The PL (Figure 5a) and CL (Figure 5b) spectra have the same energy positions and shape. The spectral dependence of the P_PL_ and P_CL_ emission are shown in Figure 5c. Similar to P_PL_, P_CL_ reaches its maximum in the low-energy spectral region. The spectral dependence of the polarization reflects the band structure of QW. It is evident that the maximum degree of polarization is achieved in the low CL (PL) photon energy region, which is formed mainly by electron transitions in the heavy-hole sub-band [23]. For higher photon energy, the PL and CL signals are the sum of electron transitions into the heavy- and light-hole sub-bands, which leads to a decrease in the CL (PL) polarization degree.

An important characteristic for spin polarimetry is the spin selectivity, where S represents the Sherman function, defined as the spin asymmetry for a 100% polarized electron beam. The detected P_CL_ in the studied QWs was 2% with the injection of a 20–25% spin-polarized electron beam [23], which provides an asymmetry (Sherman function) equal to *S_CL_* = *P_CL_/P_0_* = 0.08–0.1.

**Figure 6:**
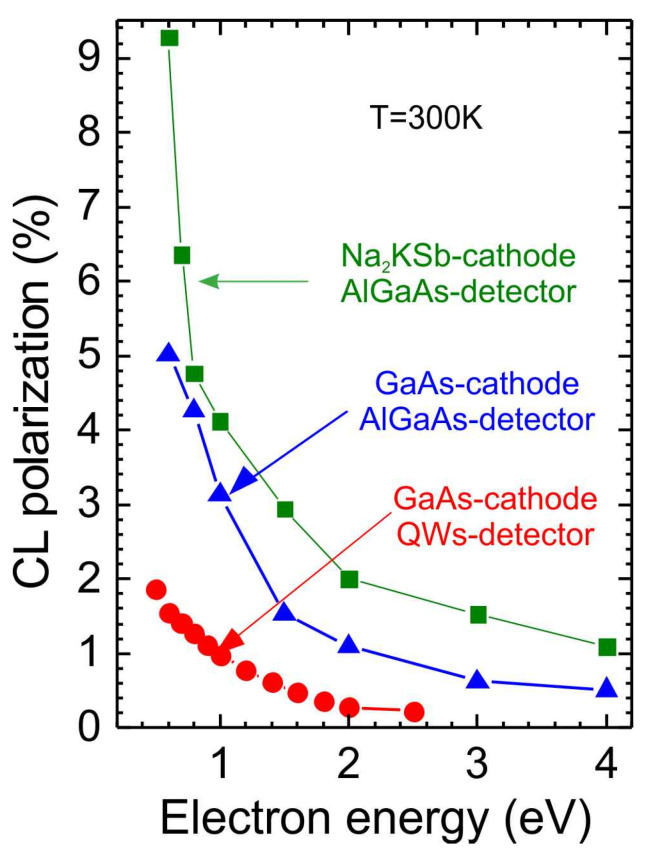
Dependence of the cathodoluminescence CP degree on the energy of injected spin-polarized electrons for the GaAs cathode and QWs detector (red circles) and Al_0.11_Ga_0.89_As detector (blue triangles) compared with the Na_2_KSb photocathode and Al_0.11_Ga_0.89_As detector (green squares). The electron energy is proportional to the bias applied between the anode and cathode, and elementary charge of electron.

For PL measurements, the energy of laser photons exceeds the splitting of light and heavy holes in QW and consequently the polarization of electrons, according to the selection rules, is 50%. [30]. Neglecting the electron depolarization inside the QW, we can estimate as *P_PL_* = 50%. One the other hand measured PL polarization is only 5% that gives similar to CL value of *S_PL_* = 0.1. Thus, for the investigated QW structure, the spin detection efficiency is about 0.1 for injected electrons of the lowest energy (Figure 1b), and it decreases with increasing electron energy, as shown in Figure 6 (red circles). It has been shown that the greatest losses in spin polarization occur in thin QW [23]. The detection efficiency can be increased by raising the spin relaxation time *τ_s_* through increase the QW thickness [31] and by decreasing the lifetime time *τ_r_* through increase in the QW doping level [30].

As a first step, we increased the thickness of the quantum well in the limit to 200 nm and used the active Al_0.11_Ga_0.89_As layer as a spin detector. The dependences of the cathodoluminescence circular polarization degree determined from spectral distribution on the electron energy in the range of 0.5–4 eV for two photocathodes (GaAs and Na_2_KSb) and two detectors (QWs and Al_0.11_Ga_0.89_As) in various combinations are shown in Figure 6. Comparing the CL polarization for QWs (red circles) and Al_0.11_Ga_0.89_As detectors (blue triangles) with the same GaAs photocathode, it can be concluded that the polarization detection efficiency, *S_CL_*, of the Al_0.11_Ga_0.89_As spin detector is 2.7 times higher than that of QWs. This means that the Sherman *S_CL_* function is 0.27 for the Al_0.11_Ga_0.89_As spin detector, which already exceeds the average Sherman function for standard Mott detectors [32]. By replacing the GaAs photocathode with Na_2_KSb [15] and keeping the Al_0.11_Ga_0.89_As target as the spin detector (Figure 6 (green squares)), we observed a further increase in the degree of circular CL polarization almost twofold, which means that the polarization of photoemitted electrons from Na_2_KSb was in the 40–50% range.

## 4. Discussion

**Figure 7:**
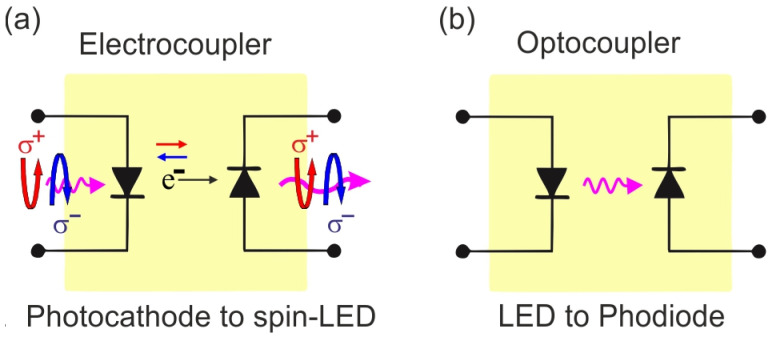
(**a**) Electrocoupler working principle. The electrocoupler can work in both current and optical modes. The optical mode ensures spin-dependent operation. (**b**) Optocoupler working principle. The optocoupler uses LED optically coupled to a photodiode (phototransistor) in a single package.

Figure 7a demonstrates the electrocoupler concept for the emission/injection of spin-polarized electrons, which incorporates the optocoupler principle (Figure 7b) and is based on signal transmission by free electrons instead of photons. The electrocoupler can work in both current and optical modes. In optical mode, the vacuum-spin LED registers the polarization of free electrons injected into semiconductor heterostructure through polarized cathodoluminescence. Currently, it is unclear whether such a device could find wide application, but it could definitely be useful for scientific applications in spin analysis methods.

The combination of two semiconductor structures separated by a vacuum gap in the vertical geometry allows us to study the physics of electron emission and injection, in particular, electron–phonon coupling during emission processes and the energy and angular distribution of the photoemitted spin-polarized electrons. One promising application could be the creation of nanophotonic photocathodes based on semiconductor nanopillar-array Mie-type resonators demonstrating quantum efficiency enhancement [33]. As a next step, lateral-type devices with spin-polarized electron emission in the plane can be considered for general electronics [3]. The practical result of this work is a compact spin polarimeter with spatial resolution, which, in combination with modern energy analyzers used in the angularly resolved photoemission method (ARPES), will make it possible to measure electron distribution by energy, momentum and all spin components, i.e., to obtain complete information on the dispersion law.

## 5. Conclusions

We fabricated a vacuum-spin LED that uses coupling between the photon helicity and the angular momentum of an electron-hole quasiparticle in the photocathode followed by emission of spin-polarized electrons and their further injection into a semiconductor heterostructure and their recombination with generation of circularly polarized light. The photoemission, injection, and detection processes of spin-polarized electrons are realized without using any magnetic material or external magnetic field. The combination of two semiconductor structures separated by a vacuum gap in the vertical geometry allows us to study the physics of emission and injection of spin-polarized electrons. The spin-current detection efficiency in spin VLED was found to be 27% at room temperature. The obtained results show that semiconductor detectors are promising for applications in spin polarimetry based on the optical detection of the spin polarization of free electrons. The created vacuum spin LED paves the way for the development of a new branch, which can be called “vacuum semiconductor spintronics”.

## 6. Patents

O.E. Tereshchenko, in Russian Patent RU 2 625 538 C1, “Spin-detector of free electrons based on semiconductor heterostructure”, 14 July 2017.

## Data Availability

Not applicable.
